# Seismic response of benchmark high-speed rail (HSR) round-ended rectangular-shaped cross-section solid (RERSCSS) concrete pier based on the shaking table tests

**DOI:** 10.1038/s41598-022-24204-7

**Published:** 2022-11-15

**Authors:** Lingun Chen, Lizhong Jiang, Xin Kang, Xiaolun Hu, Xiaoming Huang, Liang Xu, Linlin Sun, Lu Wang, Yuan Tian, Chencheng Zhai

**Affiliations:** 1 College of Civil Science and Engineering, Yangzhou University, Yangzhou, 225127 China; 2 School of Civil Engineering, Southwest Jiaotong University, Chengdu, 610031 Sichuan China; 3 Department of Civil and Environmental Engineering, University of California, Los Angeles, CA 90095 USA; 4 MOE Key Laboratory of Engineering Structures of Heavy Haul Railway (Central South University), Changsha, 410075 China; 5 Department of Civil Engineering, Central South University, Changsha, 410075 Hunan China; 6 School of Transportation, Southeast University, Nanjing, 211189 Jiangsu China; 7 School of Civil Engineering, Chongqing University, Chongqing, 400030 China; 8 CCDI (Suzhou) Exploration & Design Consultant Co., LTD, Suzhou, China; 9 Transportation Technology Development Promotion Center, China Academy of Transportation Sciences, Beijing, 100029 China; 10 Post-Doctoral Research Center, Hunan Construction Investment Group Co LTD, Changsha, 410004 China

**Keywords:** Civil engineering, Natural hazards

## Abstract

High-speed rail (HSR) has recently expanded its networks globally, but its 350 km/h bridges have not yet been tested for high-level earthquakes. This study tests the typical HSR bridge on a shaking table to assess the seismic performance in high-level earthquakes such as Maximum Considered Earthquake. Based on the model similarity theory, it creates nine round-ended rectangular-shaped cross-section solid RC HSR bridge piers. It employs the orthogonal testing method to conduct experimental design considering four influential factors: aspect ratio, axial load ratio, longitudinal reinforcement rate, and volumetric stirrup ratio. Experimental research was conducted to examine the dynamic response of these piers subjected to varying seismic impacts and design parameters, and the implications of the four factors on the seismic performance of the piers were discussed. After all the earthquake circumstances, the test findings demonstrate that the concrete of the pier specimens has not cracked or spalled much. An earthquake with a peak acceleration of 0.96 g indicates that the pier body of the standard high-speed rail round-end solid pier retains its integrity and stability. The extent of the pier's earthquake damage is not immediately evident. HSR bridges' seismic design may benefit from this research, which examines the impact of dynamic characteristics, including aspect ratio, axial load ratio, and longitudinal reinforcement rate, on HSR bridge piers' seismic performance.

## Introduction

**Figure 1:**
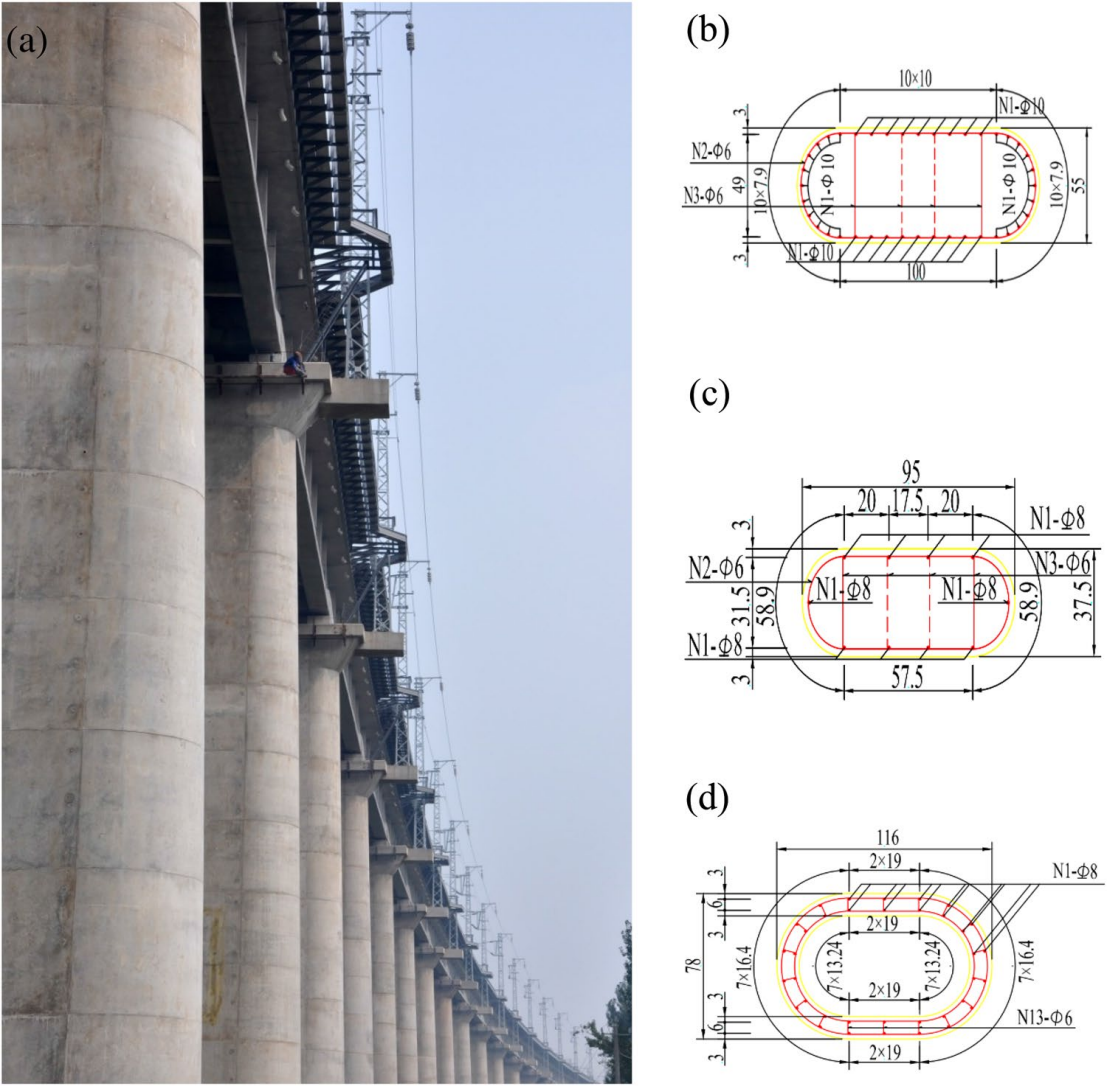
RERS cross-section piers used in HSRBs. (**a**) The bridge with RERS cross-section piers; (**b**) section of a 10 m high concrete pier, (**c**) section of an 18 m high concrete pier; and (**d**) round-ended part of a 30 m high hollow pier. Image by Dr. Lingkun Chen.

The superstructure of high-speed rail (HSR) bridges (HSRBs) is mainly box-shaped girder bridges. The HSRBs massively adopted the circular end piers to match the shape of piers and streamlined box girders, which simultaneously provides more considerable stiffness in the transverse direction and contains stress concentrations compared to those circular and rectangular sections^1–7^. Figure 1 shows the round-ended rectangular-shaped cross-section solid (RERSCSS) concrete piers used in HSR bridges. Notably, in order to meet the harsh terrain of mountainous regions, the height of these piers is often above 30 m, and their cross-section is frequently a hollow pier structure^8,9^. This article does not cover this form of the bridge pier.

Many experimental studies have been carried out to examine piers' earthquake behavior, and some of these focused on piers with rectangular sections. Han et al.^10^ built cyclic tests on five pier specimens to investigate the dynamic response of reinforced concrete (RC) bridge piers. Xia et al.^11^ conducted bi-axial quasi-static tests on 14 RC thin-walled piers, and the seismic properties were studied in detail. Chen et al.^12^ assessed the nonlinear dynamic response of HSRB with isolated bearings subjected to near-field earthquakes. Guo et al.^13^ analyzed the earthquake collapse of the HSRB piers. Also, many experimental kinds of research about the piers with a circular section. For example, Shim et al.^14^ performed quasi-static tests to investigate the seismic behavior of the precast segmental bridge piers. Yuan et al.^15^ conducted a cyclic analysis to analyze the seismic performance of circular section partially concrete-filled steel tube bridge piers.

Apart from experimental methods, validated finite element (FE) analysis has also been applied for detailed parametric analysis. For example, Guo et al.^16^ proposed a simplified mechanical model to study a self-centering bridge pier's seismic interpretation, and the quasi-static test confirmed the results. Sun et al.^17^ studied RC bridge piers' seismic behavior and established a validated FE model to study the pier's hysteretic behavior.

However, research about the seismic performance and damage of this type of RERSCSS concrete piers used in HSRB is limited. The shaking table test of the hollow pier has obtained important research conclusions^8,9^. As the loading (high-speed train) to the HSRB piers is quite different from that of conventional piers (with rectangular or circular sections), the currently available results (experimentally and numerically) studies cannot be directly applied to this typical pier for the following reasons.

Recently, more and more earthquake researchers have realized that the results, i. e., buildings' collapse resistance, are still quite skeptical after adopting the maximum considered earthquake (MCE) seismic design. Salehi et al.^18^ investigated the seismic performance of Second-Generation Hybrid sliding-rocking bridge columns through an extensive experimental study. The column specimen tested under combined lateral-torsional loading sustained minimal damage (i.e., sparse hairline cracks) under the peak drift ratios up to 2% (representing a 2475-year seismic hazard). The columns seem to have sufficient sliding capacity for intensities exceeding MCE. Another case study conducted by Li et al.^19^ is that the segmental ultra-high performance concrete bridge pier experienced lower residual deformation under the MCE event (2475-year return period). The results indicate that the segmental bridge can effectively reduce a bridge's damage probability compared to the conventional monolithic RC pier. It has resulted in the collapse probability of structures that can not be guaranteed to be less than 10% when encountering no more than MCE seismic action^20^. That is to say, even if the seismic design is based on the MCE ground motion, it is difficult to ensure that the risk level of building collapse in the United States is consistent. Throughout most municipalities of the United States, structural engineers design new buildings using the U.S.-focused IBC^21^. However, up to now, there has been little research on the influence of MCE on the seismic response of the HSRB.

In order to maintain train comfort and safety, the HSR pier's stiffness must be enhanced to decrease vibration in both directions, particularly in the cross-bridge direction. Then, the HSR pier is constructed using cross-sectional stiffness as the control index. Increasing the cross-sectional size improves bridge pier stiffness more than the longitudinal reinforcing rate. Most HSR pier has a large cross-section with limited longitudinal reinforcement. Therefore, the HSR pier differs from the light, flexible piers in buildings and bridges. This kind of pier has a low longitudinal steel ratio, low shear span ratio, and a sizeable difference between longitudinal and transverse shear span ratios. The research on the seismic performance of RERSCSS concrete piers used in HSRB mainly focuses on the direction along the bridge. The shear span ratio is generally higher than 4. The primary failure mode of piers is bending failure.

In China, after the Beijing-Shanghai HSR, with a total length of 1318 km and a speed of 350 km/h, was put into commercial operation in 2011 (the length of bridges is about 1140 km, accounting for 86.5% of the main line), the HSR entered a large-scale construction phase. Meanwhile, the HSR is likewise developing rapidly worldwide. More crucially, HSR train building started in high-intensity earthquake zones, such as China's Sichuan-Tibet Railway. The complex and changing construction and operating environment introduce numerous technical issues, yet the fast expansion of high-speed rails provides little time for researchers. The experimental research on the seismic performance of HSR circular end concrete piers is still insufficient. Therefore, specifically-targeted experimental and numerical studies are necessary for this type of RERSCSS concrete piers used in HSRB.

In order to study the seismic damage degree and characteristics of RERSCSS concrete piers under different seismic intensities, the 1/8-scaled pier specimen was fabricated and tested on the shaking table. Before carrying out this experimental study, the authors conducted extensive literature research. Notably, the shaking table experiments have been carried out at the Pacific Earthquake Engineering Research Center^22^, the University of Nevada, Reno (UNR)^23^, University at Buffalo's (UB) Structural Engineering and Earthquake Simulation Laboratory (SEESL)^24^, Railway Technical Research Institute in Japan^25^, and other joint research results based on NEES and E-Defense^26^. Undoubtedly, the research results of the researchers are of great help to the authors. During the test, the experimental system recorded the acceleration and displacement time-history curves under the peak ground acceleration (PGA) scale of 0.45 g, 0.60 g, and 0.96 g, which correspond to the frequent Chinese earthquakes (0.15 g, 0.20 g, and 0.32 g) after similarity transformation.

Moreover, the natural frequency change before and after each test scenario was also obtained. Secondly, an FE model of this specimen was also established and validated against the experiments. Using this model, more detailed seismic responses (i.e., hysteretic behavior and damage level) of the sample under these earthquakes (0.45 g, 0.60 g, and 0.96 g) were also studied. This work can benefit from understanding the seismic performance of the RERSCSS concrete piers used in HSRB and providing suggestions on their earthquake safety assessment.

## Shaking table (ST) test

### Prototype bridge

This study chose the precast-simply-supported beam bridges with a uniform span of 32 m as the prototype. The most common bridge type of HSR is a simply-supported beam bridge, and the superstructure adopts a prestressed box girder with a span of 32 m and 24 m. According to China's busiest HSR^3,4^, the length of lines using prefabricated simply-supported beam bridges is 92.08% for the Beijing-Tianjin rail, 68.24% for the Beijing-Shanghai rail, and 92.58% for Harbin-Dalian rail.

**Figure 2:**
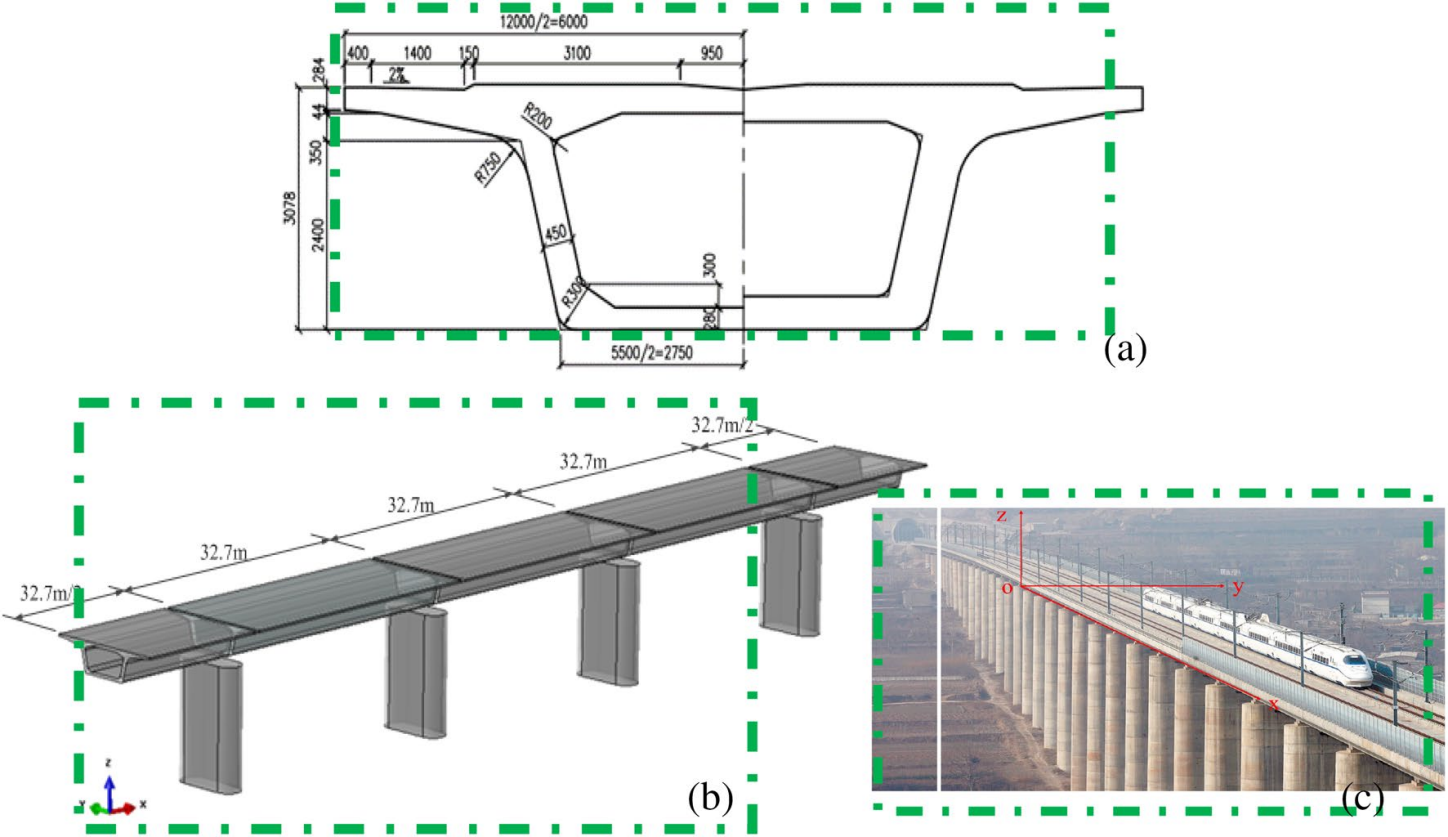
Prototype of a multi-span precast-simply-supported-beam-bridges for HSR (unit: m).

**Figure 3:**
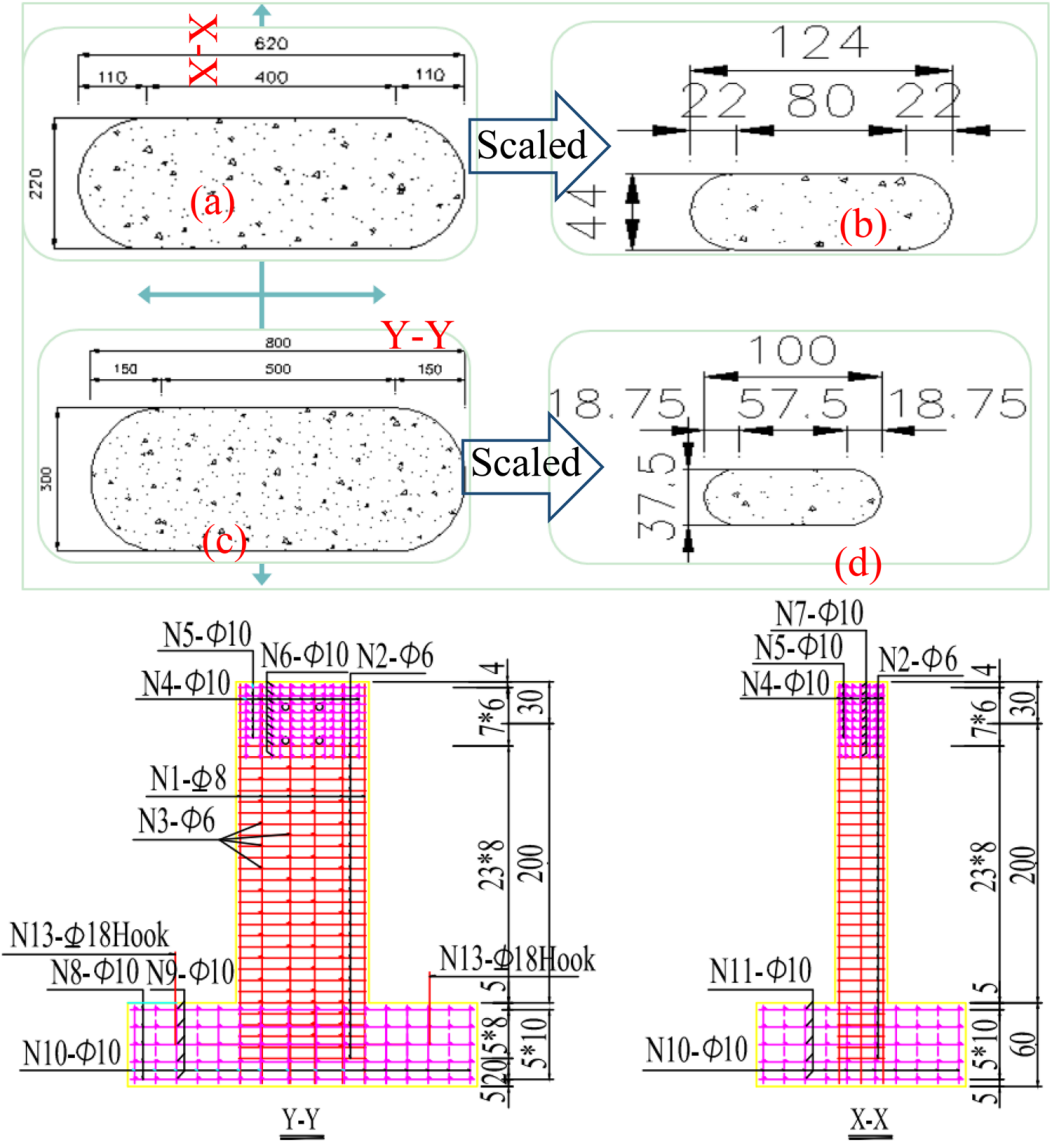
Cross-sections of RERSCSS concrete piers used in HSRB prototype and test model (unit: cm). (**a**) 8 m high pier prototype, (**b**) 8 m high pier model, (**c**) 16 m and 24 m high pier prototype, (**d**) 16 m and 24 m tall pier model.

This experimental study is based on the RERSCSS solid concrete pier of 350 km/h Passenger Dedicated Railway prepared by the China Railway Design Corporation^27^. cConsidering the variation range of the pier height of the round-ended solid piers of the typical HSR, three piers with different sizes (8 m, 16 m, and 24 m) are selected as the test prototype in this paper. The prototype bridge^27^ is shown in Fig. 2, and the cross-sections of the piers are shown in Fig. 3.

### Test design

Many studies show that the axial load ratio, the longitudinal reinforcement, the volumetric stirrup ratio, the aspect ratio, and the concrete strength significantly influence pier columns' seismic performance^2,8–10^. Because the axial load ratio of the HSR pier under the self-weight is relatively small, generally around 3%, the axial load of the pier under the earthquake will float near the axial load value under the self-weight. The axial load ratio is set within 15%.

The longitudinal steel ratio is less than 1%, and the volumetric stirrup ratio is also tiny. According to the existing research^2,8–10^, this paper determines each influencing factor's level. See Table 1 for the values of each influencing factor. The present study used the orthogonal test method to design the bridge pier model. The design parameters of the test models are shown in Table 1.

**Table 1:** Combination of variables of RERSCSS concrete piers used in the HSRB model.

Specimen No.	Height of pier (m)	Aspect ratio (l/D)	Axial load ratio (*N/f*_*c*_*A*) *%*	Longitudinal steel ratio $\rho_{{{\kern 1pt} l}}$ *(%)*	Volumetric stirrup ratio $\rho_{ v}$ *(%)*
M-1	3.0	8.0	10	0.75	0.45
M-2	3.0	8.0	15	0.15	0.15
M-3	3.0	8.0	5	0.40	0.30
M-4	2.0	5.3	5	0.75	0.15
M-5	2.0	5.3	15	0.40	0.45
M-6	2.0	5.3	10	0.15	0.30
M-7	1.6	3.6	15	0.75	0.30
M-8	1.6	3.6	5	0.15	0.45
M-9	1.6	3.6	10	0.40	0.15

Note: fc denotes the axial compressive strength of concrete; A denotes the compressive area of concrete; N denotes the axial load of the model. L is the pier height; D is the outer diameter of the round end for the M-7–M-9 model, D = 0.375 m; for the M-1–M-6 model, D = 0.44 m (Fig. 3).

In this test, the pier model is made of C35 commercial concrete (the concrete compressive strength of all specimens is 35 MPa), and the longitudinal bar of the pier model is made of a 10 mm diameter hot-rolled HRB 335 grade ribbed bar. The stirrup comprises a 6 mm diameter hot-rolled HPB 235 grade straight round bar. According to the Chinese code GB50010-2010^28^, the tensile properties of all types of reinforcement are tested.

### Pier specimens

**Figure 4:**
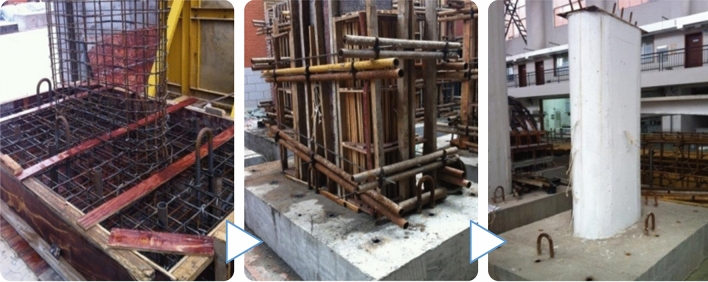
Drawings for the RERSCSS concrete piers used in the HSRB specimen (unit: cm). Top: specimen reinforcement drawing; bottom: specimen construction drawing.

Detailed sectional geometries and reinforcement arrangements of the specimen are shown in Fig. 4. The drawings of specimen reinforcement and specimen construction are shown on the top and bottom, respectively. Due to the limitation of acceleration capacity (no more than a PGA scale of 1.0 g) and size of the ST (no more than 4 (length) × 4 (width) m^2^), the prototype pier should be scaled for ST tests.

**Figure 5:**
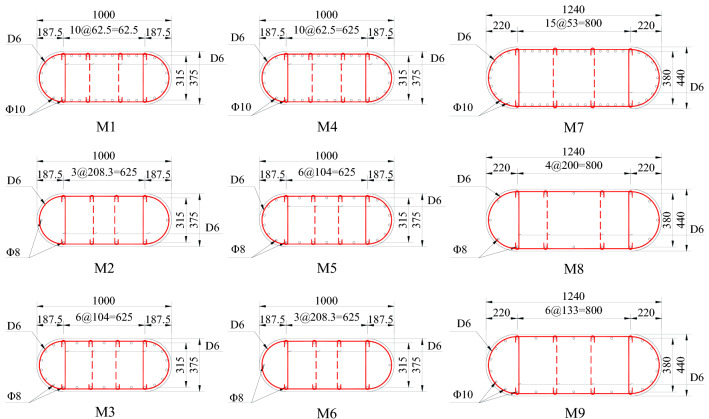
Cross-section and reinforcement arrangement of the RERSCSS concrete piers used in the HSRB test model (Note: 1. M-1–M-9 is the sample number; 2. dimension in the drawing is in cm, and the reinforcement diameter is in mm; 3. D in the picture represents HPB 235 reinforcement, and Ф serves HRB 335 reinforcement).

Therefore, an experimental specimen should be conducted for the ST test and reflects the corresponding prototype pier's dynamic features (or earthquake behavior). Figure 5 shows the cross-section and reinforcement arrangement of the pier test model.

Studies have shown that the key to designing the ST test program is determining the similar relationship between the scaled specimen and prototype^29–34^. In this study, dimensional analysis has been applied to this experiment to establish a similar relationship of physical quantities between the actual RERSCSS concrete pier and specimen. The similarity relations as shown in Table 2.

**Table 2:** Similitude parameters of high RERSCSS concrete piers used in the HSRB model.

Quantities	Similitude relation	Scaling factor	
Pier height/(m)	/	8	16/24
Length/(m)	S_l_	1/5	1/8
Modulus of elasticity/(kN/m^2^)	S_E_ = S_σ_	1	1
Stress	S_σ_	1	1
Strain	S_ε_ = 1	1	1
Density	S_σ/(_S_a ._ S_l )_	5/3	8/3
Mass/(kg)	S_m_ = S_E_S_L_^2^/S_a_	1/75	1/192
Force/(kN)	S_σ_ S_L_^2^	0.25	0.016
Frequency/(Hz)	S_L_^−0.5^ S_a_^0.5^	3.873	4.899
Acceleration/(m/s^2^)	S_a_	3	3

### Test set-up

**Figure 6:**
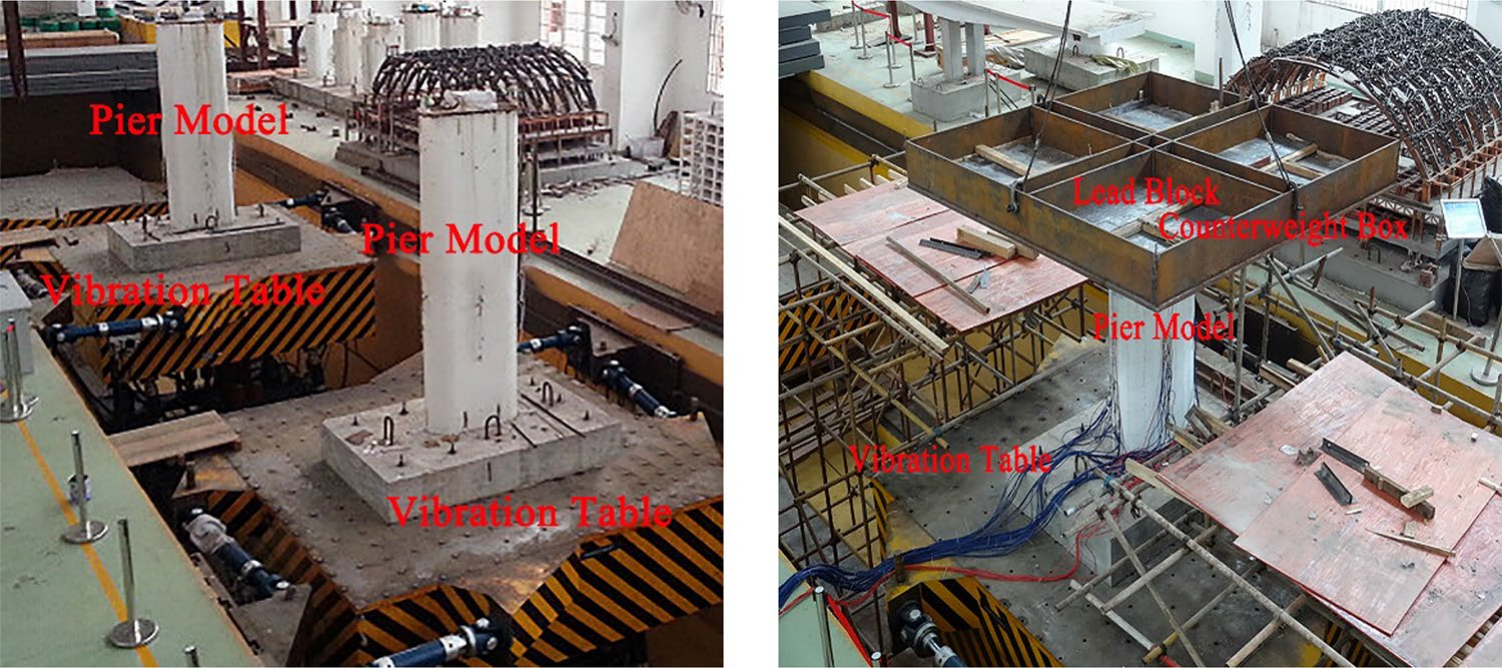
Mass locations for RERSCSS concrete piers used in HSRB ST test set-up.

Nine scaled RERSCS concrete pier specimens were tested on a shake table at the MOE Key Laboratory of Engineering Structures of Heavy Haul Railway in Central South University. The shaking table array system for the high-speed railway is shown in Fig. 6. Table 3 shows the specifications of the shaking table array system. In the test, four high-strength, finely-rolled rebar were utilized to firmly fix the model's foundation to the shaking table to avoid sliding and swaying without considering the impact of soil-structure interaction.

**Table 3:** Shaking table array system specifications.

Dimension		Capacities:	
		A table (Fixed)	B and C table (Mobile)
Table size (length × width)		4 m × 4 m	4 m × 4 m
Number of DOF		6 DOF in 3 directions	
DOF of double table linkage		12 DOF linkage	
Distance between two tables		6–25 m adjustable	
Allowable specimen payload		30 × 10^3^ kg	
Max. displacement and acceleration	Longitudinal (X)	250 mm, ± 1.0 g (full load)	
	Lateral (Y)	250 mm, ± 1.0 g (full load)	
	Vertical (Z)	160 mm, ± 1.6 g (full load)	
Vibration velocity of sine wave		750 mm/s	
Max. seismic peak velocity		1000 mm/s	
Max. overturning moment		300 kN-m	
Max. eccentric moment		200 kN-m	
Simulation frequency (max. payload)		0.1–50 Hz	

It was necessary to add mass to the top of the pier model^19^. This study simplified the prototype bridge as a single pier with a counterweight mass. According to the construction drawing and section coefficient calculation results^25^, the self-weight of a 32 m-span girder for a single-track line was 4520 kN. The secondary dead load (SDL) was equivalently considered a linear load of 60–75 kN/m, including the weight of line equipment, ballastless track structure, sidewalk support, and others. In this study, the SDL is 75 kN/m. Therefore, the lumped force exerted on the pier-top corresponding to the SDL is 1223 kN. The weight of the prototype structure's superstructure and secondary dead loads reached 5743 kN.

According to the similitude parameters of the 16 m and 24 m pier height model, the scaling factor is 1/192. Thus, the whole mass at the scaled-pier-top was 29.91 kN. A counterweight box is made and fixed on the bridge pier specimen's top. A counterweight box is made and fixed on the bridge pier specimen's top. The lead blocks are placed in the box, and the weight is 3000 kg to meet the requirements for the counterweight.

The counterweight is set to meet the similarity between the pier model's total mass and the prototype structure. The superstructure and live load of the pier are simulated by artificial weight. In other words, the prototype bridge was simplified as a single pier with a counterweight mass. The mass locations for the test set-up are shown in Fig. 6.

In order to prevent danger during the shaking table test, a steel pipe scaffold is arranged on both sides of the shaking table, and four steel wire ropes are connected with the crane hook at the top of the counterweight box to prevent the pier specimen from collapsing during the test. Then, the set-up allows large-scale models to be tested on the shake table system. The large-scale samples can then be studied without compromising the safety of the shake table system^18^.

Moreover, they can also reproduce the axial load ratio from a girder. Therefore, this arrangement can represent the prototype pier's real earthquake scenarios in the actual ST situation.

### Test instrumentations

During the shaker test, acceleration, displacement, and strain of the bridge pier model were examined, and the German IMC data gathering system was used. Four acceleration sensors (A1–A4) are suggested for the bridge pier in X and Y directions. Each pier model has four displacement sensors (D1–D4) at the bottom and top in X and Y.

**Figure 7:**
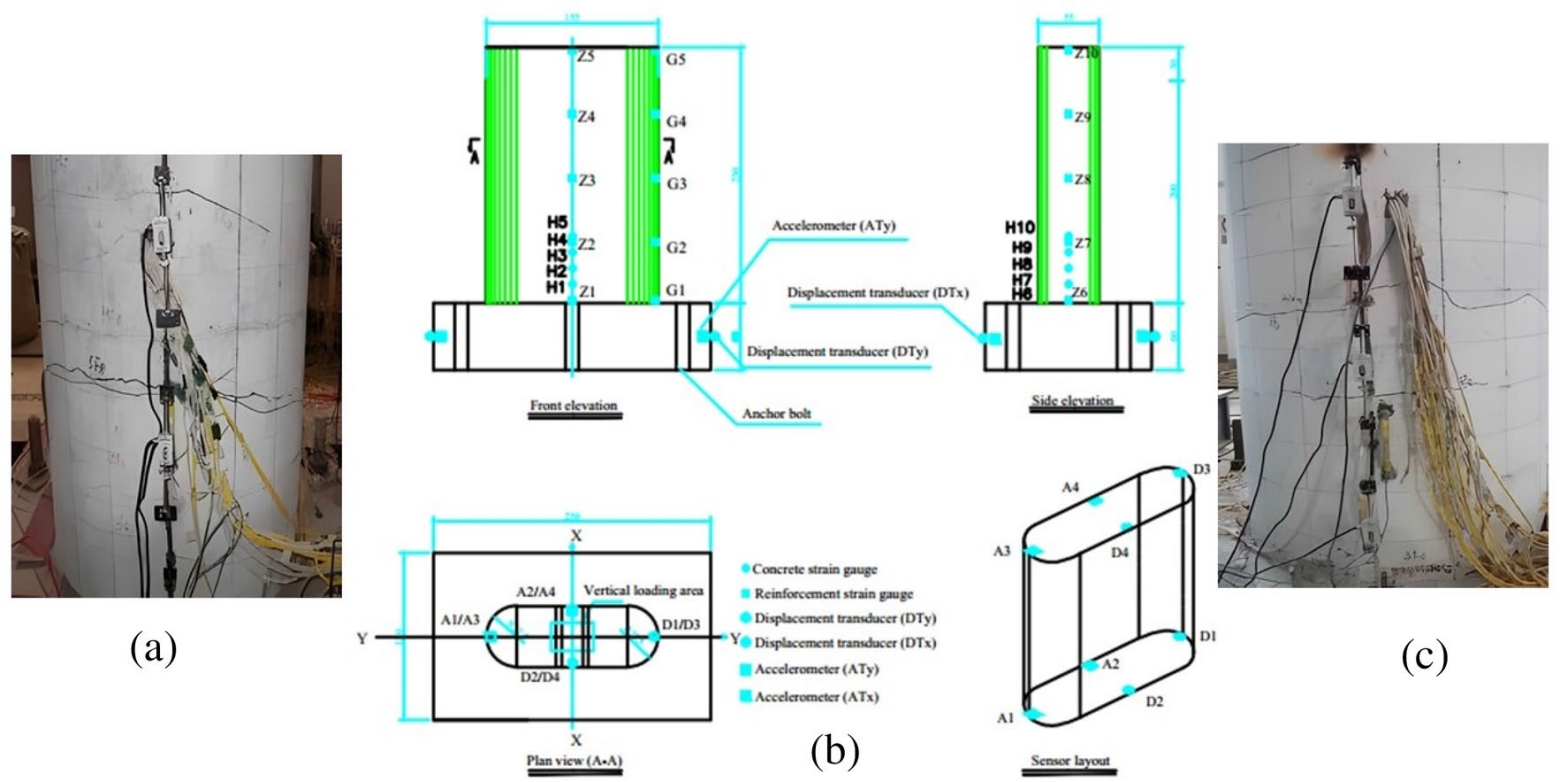
Instrumentation of data acquisition devices for experimental RERSCSS concrete piers used in HSRB specimen (unit: mm). (**a**) Test diagram of the x-direction sensor arrangement, (**b**) Schematic diagram of the sensor arrangement, and (**c**) test diagram of the sensor arrangement in y-direction.

Strain sensors were placed on the longitudinal reinforcement, hoop, and concrete on the pier specimen to monitor their strain states under different earthquake circumstances. In this test, 20 longitudinal strain gauges were arranged on each pier model, numbered Z1–Z5 (Z11–Z15) in the cross-bridge direction (−y) and Z6–Z10 (Z16–Z20) in the longitudinal direction (−x); 5 hoop strain gauges were numbered G1 to G5; 20 bow strain gauges were used to test concrete strains. The 20 bow strain gages for concrete strain testing are numbered from bottom to top as H1–H5 (−y) and H6–H10 (L16–L20) in the longitudinal direction (−x). The strain sensor arrangement is shown in Fig. 7b. The test photos are shown in Fig. 7a and c.

### Input motion and seismic hazard levels

The bridge pier specimens are based on an assumed prototype in a region of high seismicity, located in the seismic fortification intensity of the 8-degree zone^28,35^. This work utilized three seismic accelerations, 0.30 g, 0.60 g, and 0.96 g, to simulate bridges in various seismic zones.

According to the Chinese Code for Seismic Design of Railway Engineering (GB50111-2006)^28^ and the Standard for Classification of Seismic Protection of Building Construction (GB 18306-2015)^35^, the national seismic fortification range is divided into seismic intensities of 7, 8, and 9 degrees, meaning different seismic fortification criteria and design earthquake intensities apply to different seismic fortification ranges. It can be seen from Table 3 that the different 7 and 8-degree seismic partitions include two seismic accelerations, respectively, which are considered the effects of near-field earthquakes.

The motions that were used for the shake table tests were calculated based on the 1994 Northridge-01 earthquake as recorded from the ground station at the City Hall of Santa Monica city with a maximum PGA of 0.93 g. The earthquake motion was scaled to various seismic hazard levels. Theoretically, railroad bridges are more concerned with transverse vibration control to ensure train safety. Based on this assumption, the piers are built with substantially higher transverse stiffness than longitudinal stiffness. Second, according to the Chinese Code for Seismic Design of Railway Engineering (GB50111-2006)^28^ Article 7.1.4, the seismic bridge test must compute horizontal seismic activity in the cis-bridge and cross-bridge directions during the design phase. For a 9-degree earthquake, the cantilever and rigid main structure must also account for vertical seismic activity. All specimens were loaded in -x and -y directions and vertical seismic effects were ignored.

Three seismic hazard levels were considered in this study according to the Code for Seismic Design of Railway Engineering GB50111-2006^28^, i.e., the design primary earthquake (DBE) and the MCE event, respectively^20^. As dictated in the Table 4. The time axis of the prototype motions was compressed by a factor of $\sqrt {1/15} { = 1/3}{\text{.873}}$ (for the 8 m pier height model) and $1/\sqrt {24} = 1/4.899$ (for the 16 m and 24 m pier height to account for the model's scale. The acceleration amplitude was scaled by 3 to study the piers' damage evolution under different seismic excitations' intensities.

**Table 4:** Earthquake intensity of different seismic zones for different seismic codes (g).

Specification	Seismic level					
	Recurrence period (years)	7-degree zone		8-degree zone		9-degree zone
GB50111-2006^28,35^	50 (Frequent Earthquake )	0.04	0.05	0.07	0.10	0.14
	475 (Design Basis Earthquake)	0.10	0.15	0.20	0.30	0.40
	2475 (High-level Earthquake)	0.21	0.32	0.38	0.57	0.64
IBC 2018^20^/ASCE/SEI 7–10^36^	2475 (Maximum Concerned Earthquake) (S_S_)	0.65	1.01	1.03	1.38	1.58

The values underlined are the input accelerations for the shaking table tests.

**Figure 8:**
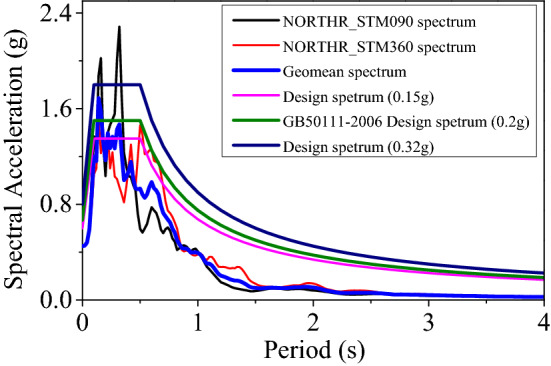
Response spectra at all considered hazard levels.

The motion was selected so that the geometric mean spectrum of the horizontal acceleration components of the ground motion ensembles used for testing matched the response spectrum of the Code for Seismic Design of Railway Engineering GB50111-2006^28^ in the period range of interest (approximately 0.085 s–0.335 s in this study). The corresponding 5% damped acceleration response spectra for all hazard levels are presented in Fig. 8. All earthquake motions were scaled to these hazard levels by multiplying the acceleration values' amplitude values by a corresponding scalar. i.e., the test motions were normalized to 0.15 g and 0.20 g for DBE, and 0.32 g for MCE event.

Secondly, after the similarity transformation, the experimental earthquake intensity PGA was a scale of 0.45 g, 0.60 g, and 0.96 g, respectively. They correspond to simulate the actual earthquake excitation of 0.15 g (approximately corresponding to OBE level), 0.20 g (corresponding to DBE level), and 0.32 g (corresponding to MCE level). It is worth noting that the shake table's acceleration limit is no more than 1.00 g. The maximum intensity of the experimental earthquake was selected as 0.96 g for this test. According to the similarity ratio, the specific operation determines the seismic hazard level according to the response spectrum and then adjusts the amplitude.

### Shake table test protocol

Table 5 gives the details of the test scenarios. In this test, The Santa Monica, City Hall Ground wave from the 1994 Northridge earthquake was selected for shake table tests [2,36,37]. As mentioned above, the prototype bridge in this study is the most widely used bridge type. Therefore, without generality loss, the unscaled ground motion's magnitude assumed that the unscaled ground motion simulated in the shake table tests was 6.4–7.9. The site was supposed to be soil with Vs-30 = 360–500 m/s per seismic design criteria of the Code for Seismic Design of Railway Engineering GB50111-2006^28^, Soil Profile Type B/C boundary. It was assumed that an angle of 90°component of the motions was selected to maximize the y-direction bridge's in-plane rotation.

**Table 5:** Sequence of the ST test scenarios.

Test scenario	Prototype intensity	Test intensity (g)	
		x-direction	*y*-direction
1	White noise	0.05	0.05
2	0.15 g	0.45	NA
3		NA	0.45
4	White noise	0.05	0.05
5	0.20 g	0.60	NA
6		NA	0.60
7	White noise	0.05	0.05
8	0.32 g	0.96	NA
9		NA	0.96
10	White noise	0.05	0.05

**Figure 9:**
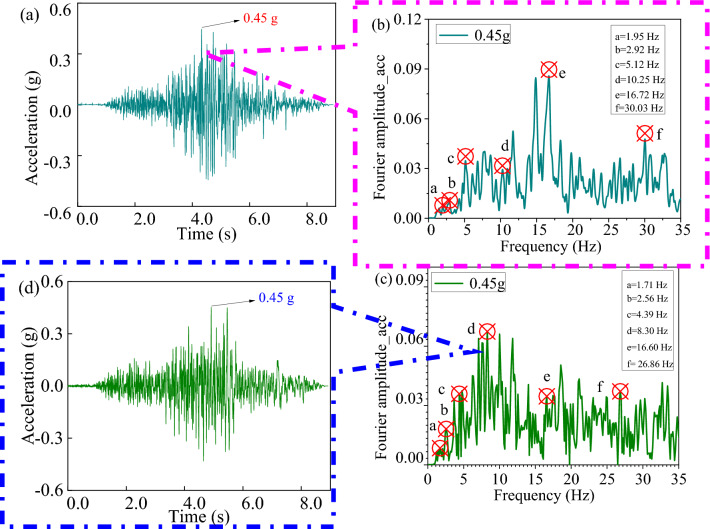
Northridge waves used for the ST test (**a**) in the *x*-direction; (**d**) in the *y*-direction; (**b**) Fourier spectrum of the earthquake wave for the *x*-direction; and (**c**) Fourier spectrum of the earthquake wave for the *y*-direction.

An example of the recorded time history and spectrum for the 0.45 g PGA earthquake (in the x and y directions) is illustrated in Fig. 9a and b, respectively. Figure 9c and d show the spectrum characteristics of ground motion. It can be observed that the dominant frequency of the x-direction is 10.25 Hz, and the dominant frequency of the y-direction is 8.30 Hz. Moreover, the specimen was also subjected to low-amplitude white noise excitation (PGA scale of 0.05 g) to monitor dynamic characteristics changes before and after being subjected to seismic excitations.

## Test results and discussion

### Experimental observation

In this study, nine large-scale RERSCSS concrete piers used in HSRB models are built and manufactured for shaking table testing using various design parameters, including axial load ratio, longitudinal reinforcement rate, volumetric stirrup ratio, aspect ratio, and pier height. The post-earthquake damage state of bridge piers is observed, and the dynamic characteristics, acceleration, displacement, strain, and hysteresis curves of the RERSCSS concrete piers used in HSRB models are analyzed under various levels of ground shaking to investigate the seismic performance of solid high piers used in railroad bridges.

**Figure 10:**
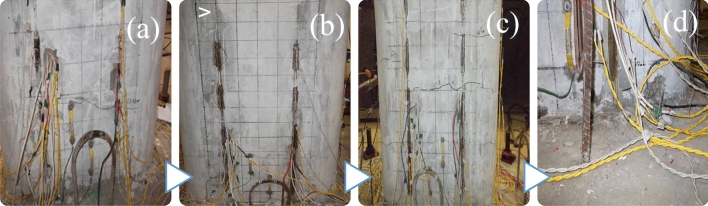
Damage at the bottom of the M-1 model during the experimental program. Height (2.0 m); Aspect ratio 8.0; lxial compression ratio (10%); longitudinal reinforcement rate (0.75%); volumetric volumetric stirrup ratio (0.45%). (**a**) First crack on the north side; (**b**) first crack on the south side; (**c**) final crack on the north side; (**d**) concrete crush damage.

Figure 10 shows the test phenomena of the M-1 model during the experimental program. The test phenomena reveal that for pier models with 16 m and 8 m pier heights, the crack at the foot of the pier is not evident during a 0.32 g (High-level Earthquake in the 7-degree zone) earthquake; Nevertheless, the pier model exhibits obvious crack when the pier height is 24 m.

### Natural frequency

This section analyses the post-seismic damage state of the specimen subjected to each experimental earthquake scenario. One method is to compare the change in its fundamental frequency. The shift in specimen stiffness can be reflected by the decrease in frequency (correctly $\omega = \sqrt {K/M}$). Therefore, to obtain the transformation of the natural frequency of the specimen between each test, a white noise excitation of 0.05 g PGA was applied to excite the model both before and after the earthquake excitation. The sampling frequency (*f*_*s*_) and white noise (*T*) were 500 Hz and 120 s.

**Figure 11:**
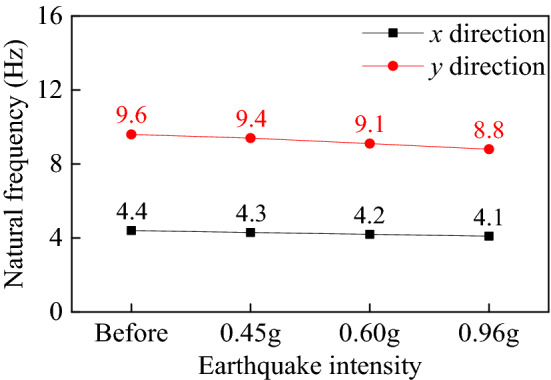
Fundamental frequencies of the pier specimen M-1 subject to earthquake actions.

After applying the Fast Fourier Transform (FFT) to the recorded acceleration time histories, the pier specimen's natural frequency was obtained. Figure 11 illustrates the M-1 sample's fundamental frequency under earthquakes in both *x* and *y* directions. It shows clearly that the change in natural frequency is insignificant.

Specifically, the fundamental frequency cumulatively changed from about 4.4 Hz (before any seismic excitation) to 4.1 Hz (after the 0.96 g earthquake excitation) in the *x*-direction and from around 9.6 Hz to 8.8 Hz in the *y*-direction. Therefore, It is reasonable to conclude from the experimental results that this specimen remains elastic after being subjected to all the observed excitations (i.e., 0.45 g, 0.60 g, and 0.96 g) and can withstand more severe earthquakes excitations. Namely, the so-called MCE_R_ hazard exceeds 0.96 g for the scaled pier specimens or 0.32 g for the prototype pier (linked to the similarity relationship, Table 5). Moreover, the results show that the attenuation degree of the natural frequency of the bridge pier in the x-direction is more significant than that in the y-direction; that is, the pier is more prone to damage in the x-direction (longitudinal direction) subjected to the earthquake action.

**Figure 12:**
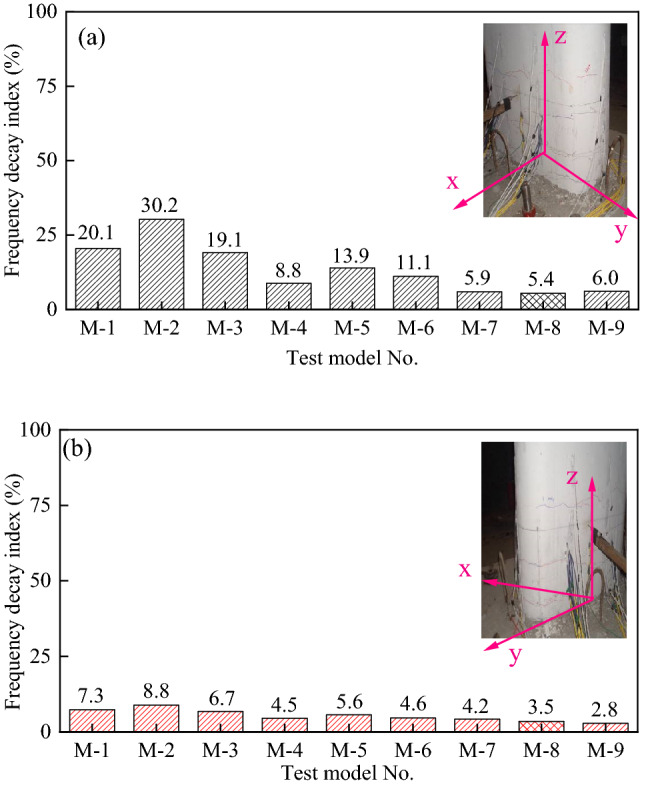
Attenuation of the fundamental frequency of post-earthquake pier specimens. (**a**) x-direction, (**b**) y-direction.

It is essential to understand what happens to each pier model's fundamental frequency (f) when exposed to seismic activity; therefore, the cumulative attenuation of the fundamental frequency (Δ*f*) is shown in Fig. 12. While Δ*f* = (*f*_W.O._– *f*_0.96 g_)/*f*_W.O._ × 100%, *f*_W.O._ represents the frequency of the bridge pier when not exposed to seismic activity, and *f*_0.96 g_ is the frequency of the bridge pier after the highest intensity of 0.96 g seismic activity.

Findings from this research indicate that there is more attenuation of the bridge pier's self-oscillation frequency (x-direction) than in the y-direction, which means that the bridge piers are a more significant threat to earthquake damage in the x-direction (along with the bridge).

Another factor that makes seismic damage more likely is that the higher the pier rises (M-1, M-2, and M-3, 3 m), the more pronounced the cumulative attenuation of its fundamental frequency becomes (about 20–30% in the x-direction, about 7% -8% in the y-direction). Conversely, the shorter pier (M-7, M-8, and M-9) has less frequency attenuation (about 5–6% in the x-direction, about 3–4% in the y-direction).

Similarly, a larger axial load ratio for identically-sized piers results in greater pier fundamental frequency attenuation (e.g., models with 15% axial load ratio: M-2, M-5, and M-7).

The height of the pier and the axial load ratio to the height of the pier significantly impact how much earthquakes attenuate the fundamental frequency of bridge piers. The influence of reinforcement rate and hoop rate is not apparent.

### Acceleration responses

**Figure 13:**
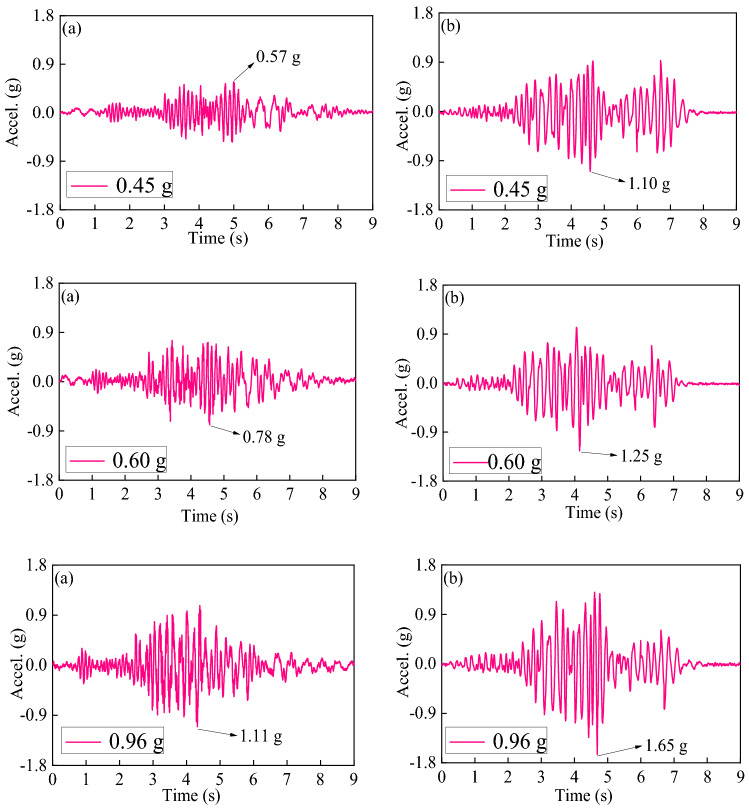
Time-history of acceleration at the top of M-1 pier specimen under different PGA. (**a**) x-direction, (**b**) y-direction.

**Figure 14:**
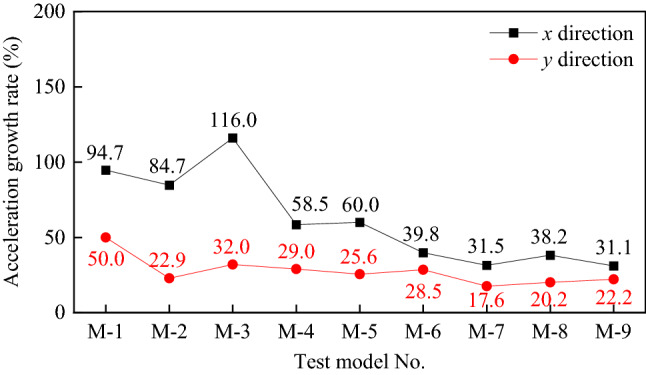
Growth rate of acceleration at the top of each bridge pier with seismic intensity.

This section examines the peak acceleration response of the bridge pier test model. Figure 13 depicts the peak acceleration time curves of the bridge pier model M-1 in the cis-bridge and cross-bridge directions. Figure 14 shows the growth rate of acceleration at the top of each bridge pier with seismic intensity. The acceleration growth rate at the top of the bridge pier ***GR***_***A***_ = ((*A*_*0.96*_*-A*_*0.45*_) /*A*_*0.45*_) × 100% is described here to characterize the variation of acceleration. *A*_*0.96*_ denotes the acceleration reaction at the pier top for a PGA of 0.96 g, and the comparison follows.

The tests show that the acceleration response at the top of the pier increases with the seismic input intensity (0.45 g to 0.96 g), but the acceleration growth rate decreases with the decrease of pier height.

Firstly, the test findings demonstrate that the acceleration at the bridge pier's top rises with pier height reduction, and the acceleration response in the cross-bridge direction is more significant than that in its cis-bridge direction. When tested with a 0.45 g test earthquake, the maximum absolute acceleration for high piers (M-1, M-2, and M-3, 3 m) was about 0.50 g ~ 0.72 g down-bridge and 1.00 g ~ 1.53 g cross-bridge; while for short piers (M-7, M-8, and M-9, 1.6 m), the maximum absolute acceleration was about 1.23 ~ 1.43 g and 1.68 ~ 1.88 g, respectively.

Second, for a given pier height, a lower axial load ratio results in a faster rate of acceleration increase. For example, M-3 (3 m, 5% axial load ratio), M-5 (2 m, 5% axial load ratio), and M-8 (1.6 m, axial load ratio 5%). In addition, the relationship between longitudinal reinforcement rate and the volumetric stirrup ratio is not immediately apparent.

As shown in Fig. 14, the acceleration growth rates of tall piers (M-1, M-2, and M-3, 3 m) ranged from about 84.7% to 116.0% in the down-bridge direction and from about 22.9% to 50.0% in the cross-bridge direction, while the acceleration growth rates of short piers (M-7, M-8, and M-9, 1.6 m) with seismic intensity ranged from about 31.1% to 38.2% in the down-bridge direction and from about 17.6% to 22.2%.

For the most part, the axial load ratio and longitudinal reinforcement rate have a little discernible effect on the acceleration response of bridge piers during an earthquake. The pier height, however, does have a significant impact.

### Displacement responses

**Figure 15:**
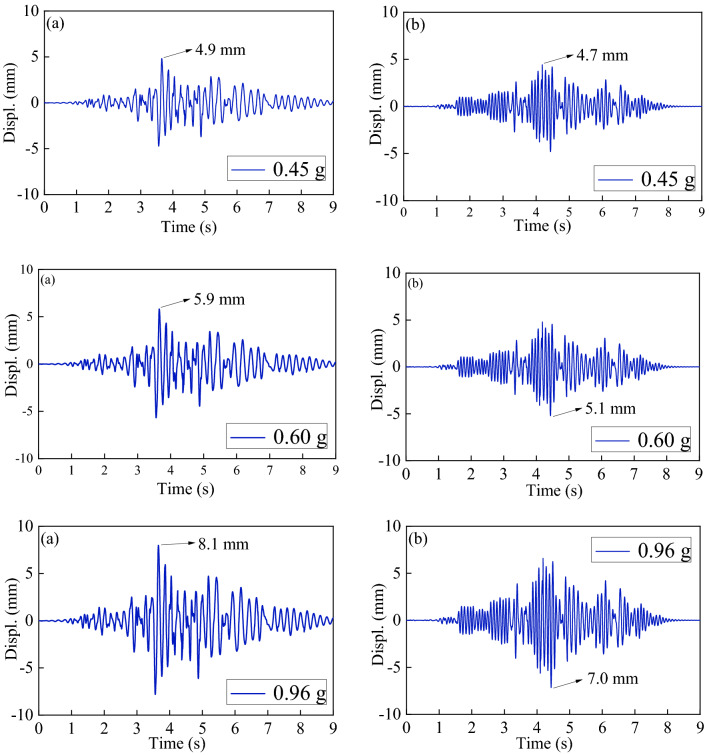
Time-history of displacement at the top of pier model M-1 under different PGAs, (**a**) down-bridge direction, (**b**) cross-bridge direction.

These bridge piers' seismic displacement responses are examined in this section using shaking table test data. For example, the time-history curves of the pier top displacement under all seismic circumstances for the pier model M-1 are shown in Fig. 15.

**Figure 16:**
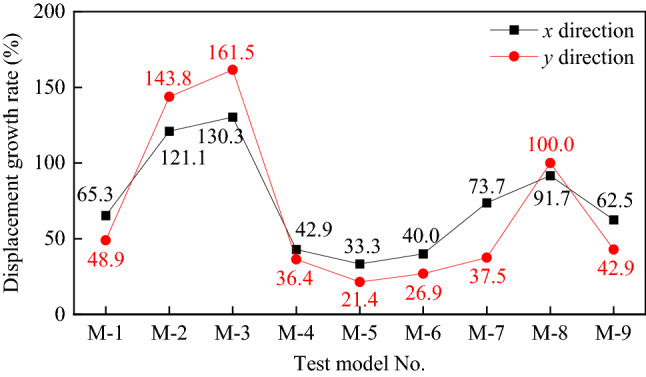
Growth rate of model displacement with seismic intensity for each bridge pier.

The test findings demonstrated that, it was found that the higher up a pier was, because its cross-sectional stiffness (EI_y_) in the y direction of the bridge was more significant than in the x direction of the bridge (EI_x_), The displacement of the pier's top in the longitudinal (-x) direction of the bridge is higher than its displacement in the transverse (-y) direction of the bridge. Figure 16 shows the displacement growth rate ***GR***_***D*=**_((*D*_*0.96*_*-D*_*0.45*_)/*D*_*0.45*_) × 100%. There is a clear correlation between height and displacement of piers, which is even stronger when the piers are located in the y direction of the bridge, i.e., EI_y_ > EI_x_, the displacement of the pier top under an earthquake is also more significant in the y direction than in its x direction.

Second, the lower the axial load ratio for the same pier height, the greater the displacement increase rate for the same height pier. It is not clear how the longitudinal reinforcement rate and volumetric stirrup ratio affect the axial load ratios of M-3 (3 m), M-5 (2 m), and M-8 (1.6 m; axial load ratios of 5%).

In summary , the pier's displacement reaction during an earthquake seems to be more strongly influenced by its height, followed by the axial load ratio, than the longitudinal reinforcement rate and the e volumetric stirrup ratio.

## Hysteresis behavior

The hysteresis curve is the load-deformation curve obtained under reciprocal force cycles. It reflects the structure's deformation characteristics, stiffness degradation, and energy consumption during repeated stresses and serves as the basis for determining the restoring force model and conducting nonlinear seismic response analysis.

**Figure 17:**
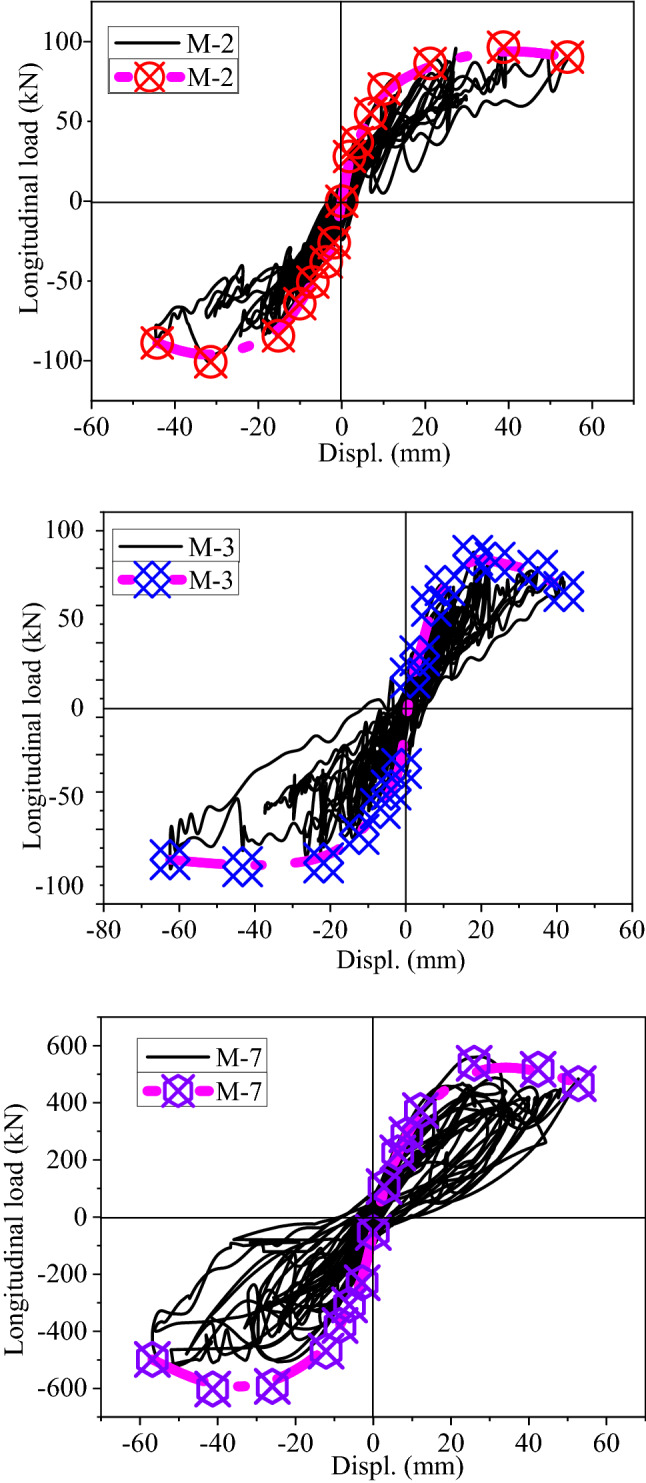
Load–displacement hysteresis curves for bridge pier model in the longitudinal direction.

Figure 17 shows the load–displacement hysteresis curves for the bridge piers model in the longitudinal direction. The test findings indicate that the longitudinal reinforcement rate significantly affects the form of the bridge pier model's hysteresis curve. When the longitudinal reinforcement rate of the pier model is 0.15% (M-2, M-6, and M-8), the hysteresis curve exhibits prominent pinching features, and the single hysteresis loop is S-shaped, with a small area and low energy consumption. When longitudinal reinforcement is applied at a rate of 0.4% (M-3, M-5, and M-9), the hysteresis curve remains pinched, the difference between the unloading and loading curves becomes apparent, and the pier's energy dissipation performance slightly improves when longitudinal reinforcement is applied at a rate of 0.15%. When the longitudinal reinforcement rate is 0.75% (M-1, M-4, and M-7), the pier model's hysteresis curve becomes full, and its energy dissipation performance is superior to that of other models.

Numerous studies have shown that factors such as the aspect ratio (height of pier), axial load ratio, longitudinal steel ratio, and volumetric stirrup ratio greatly impact the seismic performance of piers and columns. The key factors influencing the HSRB pier seismic performance have been presented in Table 2 and are shaded. Since the volumetric stirrup ratio of the model is low, the authors selected M-2, M-3, and M-7 for comparison to analyze the influence of hysteretic energy dissipation. Figure 13 shows that the longitudinal reinforcement rate has a greater influence on the shape of the hysteresis curve, and the energy dissipation capacity increases with increasing the longitudinal reinforcement rate.

## Conclusions

In this paper, nine typical RERSCSS RC piers are designed and tested using the orthogonal test method on large shaking table piers. Three piers have a scaling ratio of 1/5 (corresponding to 8 m pier height), three piers have a scaling ratio of 1/8 (corresponding to 16 m pier height), and three piers have a scaling ratio of 1/8. (corresponding to 24 m pier height). Test results were compared and discussed to analyze the dynamic response of different design parameters: aspect ratio, axial load ratio, longitudinal steel ratio, and volumetric stirrup ratio on the dynamic response of these piers under rare earthquakes, including crack development, acceleration, and displacement response, and hysteresis characteristics of the piers. The following conclusions have been drawn from this investigation's experimental and analytical findings.

1. There was no substantial cracking or spalling of the concrete in the bridge pier specimens after all seismic conditions. It indicates that the pier of a typical Chinese HSR round-end solid pier still retains excellent integrity and stability under the earthquake with a peak acceleration of 0.32 g (seven degrees uncommon), and the earthquake damage to the pier is not significant.
2. For high piers (3.0 m, M-1, M-2, and M-3), the fundamental frequency in the x-direction was around 4.3 Hz in the absence of seismic influences, and about 11.5 Hz for the shorter piers (1.6 m, M-7, M-8, and M-9). As an earthquake's strength increases, each pier specimen's fundamental frequency slowly decreases because the damage to the test specimen caused by the earthquake makes the pier less stiff.
3. Compared to the short piers (1.6 m high, specimens M-7, M-8, and M-9), the pier top displacement growth rate of the high piers (3.0 m high, specimens M-1, M-2, and M-3) is significantly greater, and the piers are more susceptible to plastic deformation, while the effect of the longitudinal steel ratio, the volumetric stirrup ratio, and the axial load ratio on displacement is not evident.
4. As the transverse reinforcement rate of railroad bridge piers is limited, the longitudinal steel ratio has a higher effect on the hysteresis curve of the pier model. When the longitudinal reinforcement rate of the model is 0.15%, the hysteresis curve pinching characteristics are quite obvious. When the longitudinal reinforcement rate increases to 0.75%, the hysteresis curve of the bridge pier model become relatively full, and the energy dissipation performance is better than other models.
5. Note that although the HSR piers were built according to the current seismic code, excessive lateral displacement is not permitted for train safety passing on the bridge. Consequently, the HSR piers cannot serve as either capacity or ductile members. As such, this is one of the goals of the shaking table test used on piers for high-speed rail lines.

## Data Availability

The authors declare that all relevant data are available within the article.
